# Photons to Formate: A Review on Photocatalytic Reduction of CO_2_ to Formic Acid

**DOI:** 10.3390/nano10122422

**Published:** 2020-12-04

**Authors:** Hanqing Pan, Michael D. Heagy

**Affiliations:** Department of Chemistry, New Mexico Institute of Mining and Technology, Socorro, NM 87801, USA; hanqing.pan@student.nmt.edu

**Keywords:** CO_2_ reduction, formate, photocatalyst

## Abstract

Rising levels of atmospheric carbon dioxide due to the burning and depletion of fossil fuels is continuously raising environmental concerns about global warming and the future of our energy supply. Renewable energy, especially better utilization of solar energy, is a promising method for CO_2_ conversion and chemical storage. Research in the solar fuels area is focused on designing novel catalysts and developing new conversion pathways. In this review, we focus on the photocatalytic reduction of CO_2_ primarily in its neutral pH species of carbonate to formate. The first two-electron photoproduct of carbon dioxide, a case for formate (or formic acid) is made in this review based on its value as; an important chemical feedstock, a hydrogen storage material, an intermediate to methanol, a high-octane fuel and broad application in fuel cells. This review focuses specifically on the following photocatalysts: semiconductors, phthalocyanines as photosensitizers and membrane devices and metal-organic frameworks.

## 1. Introduction

The sun provides 100,000 TW of energy in one hour that is enough to power the earth for one year, [1] but the energy needs to be storable and transportable (i.e., chemical fuel) because renewable energy sources generally provide an intermittent supply of energy [2]. In principle, all terrestrial energy sources, such as fossil fuels and uranium, can be considered as a solar fuel; it just depends on the time frame. For the purpose of this review, when solar energy is converted into chemical fuel on a diurnal basis the term solar fuels applies. As a mitigation strategy, C1 sources such as CO_2_ or its pH neutral dissolved version, bicarbonate become concentrated energy sources for long-term storage capacity via energy input from solar irradiation [3]. In 2019, fossil fuels still supply 84% of the world’s energy [4]. In addition to limitations in the availability of fossil fuels, the emission of greenhouse gases, especially carbon dioxide, upon the combustion of fossil fuels is a major contributor to rising levels of anthropogenic carbon dioxide in the atmosphere. With the limitations in availability of fossil fuels, new sources of energy that provide a large-scale, sustainable energy supply must be developed. Solar energy is the most abundant renewable energy source available that can meet our future energy demands [5]. One potential approach towards generating renewable fuels is to use solar energy to directly reduce atmospheric or locally produced CO_2_ to liquid fuels. This approach, referred to as “chemical carbon mitigation” can lead to methanol as an end product, a useful solar fuel. The concept of “the methanol economy,” championed by Chemistry Nobel laureate George Olah, highlights methanol as a renewable and readily transportable fuel and an alternative to the hydrogen economy. A key component to several C1 conversion methods in the methanol economy is the solar-driven conversion of carbon dioxide to formic acid and ultimately to methanol, a renewable and regenerative C1 fuel [6,7,8].

The photocatalytic reduction of CO_2_ is a multi-electron transfer process that can lead to the formation of many different products depending on the reaction pathway and the number of electrons transferred. Two-electron products include carbon monoxide, formic acid and oxalate. Methanol is the six-electron product and methane is the result of eight electron transfers [9,10,11,12,13]. The reduction half-reaction utilizes the photogenerated electrons, leaving behind the photogenerated holes. Ideally, the oxidation half-reaction would lead to the oxidation of water generating hydrogen or oxygen but very few systems can accomplish simultaneous oxidation and reduction reactions [9]. Therefore, research has focused on developing a sacrificial agent that can efficiently scavenge the photogenerated holes.

**Figure 1:**
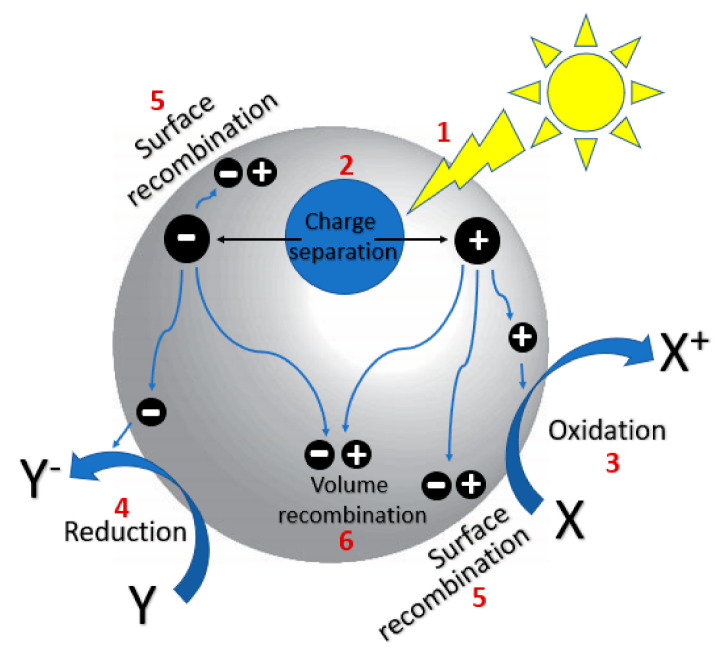
Photo-excitation of a semiconductor and subsequent generation of charge carriers and decay pathways. X = electron donor, Y = electron acceptor.

**Figure 2:**
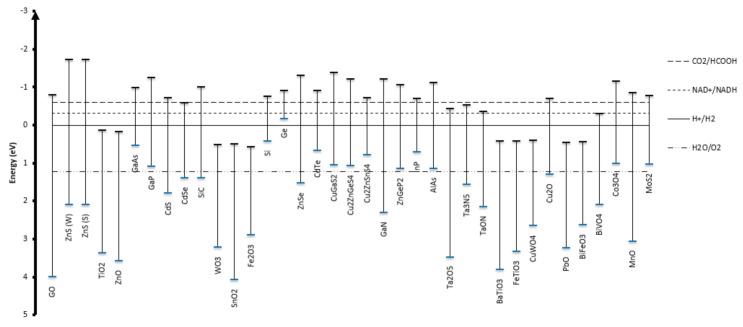
Valence and conduction band edge potentials of various semiconductors, along with reduction potentials for CO_2_/HCOOH, NAD^+^/NADH, H^+^/H_2_ and H_2_O/O_2_.

Photocatalysis follows three key steps (Figure 1): (1) Photon absorption by semiconductor, (2) charge separation, (3) surface reactions. Semiconductors are attractive materials for photocatalysis because their band gap energies are similar to that of UV or visible light (Figure 2) [9]. Upon absorption of a photon with energy greater or equal to the band gap of the semiconductor, an electron is excited from the valence band to the conduction band, leaving behind a hole. The photo-generated charge carriers then migrate to the semiconductor surface and transfer to the adsorbed molecules, which initiates the subsequent oxidation or reduction reactions. The formation of charge carriers is followed by several de-excitation pathways (Figure 1). Transfer of the charge carrier, either electron or hole, to the acceptor molecules leads to its oxidation or reduction (pathway 3 and 4). The carriers can recombine with the opposite charge carrier trapped at the surface (pathway 5) or recombination can occur in the bulk of the semiconductor (pathway 6). Three major drawbacks of photocatalysis include (1) narrow region of optical absorption, (2) rapid electron/hole recombination and (3) lack of understanding of photocatalysis mechanisms. This review aims to address these challenges.

This review is structured as follows: Section 2 and Section 3 discusses the fundamentals of photocatalytic CO_2_ reduction and explains why our work is focused on photocatalytic bicarbonate reduction to formate. Section 4 divides the catalysts into semiconductors, phthalocyanine-semiconductors and metal-organic frameworks. Lastly, Section 5 discusses the outlook of photochemical CO_2_ reduction to formate and Section 6 summarizes the review with concluding remarks.

## 2. Fundamentals of Photocatalytic CO_2_ Reduction

### Thermodynamics of CO_2_ Reduction

Photocatalytic reduction of CO_2_ has a positive ΔGº value, meaning the process is non-spontaneous and requires significant energy input from the incident photons. As mentioned previously, methanol production from CO_2_ reduction requires six protons and six electrons. Recently, there are studies in which homogeneous sensitizers coupled with metal co-catalysts have been shown to produce only CO and formic acid [14]. This observation of the incomplete six-electron reduction to methanol is governed by thermodynamics. Methanol production in an aqueous medium at pH 7 occurs at a slightly more positive potential (−0.39 V vs. NHE) than formate (−0.58 V vs. NHE) (Table 1). In addition, reduction of CO_2_ to formate only requires two electrons whereas reduction to methanol requires six.

Since carbon is in its highest oxidized state in CO_2_, its reduction can lead to a wide variety of products (CO, CH_4_, CH_3_OH, HCOOH and many other products). CO_2_ is a thermodynamically stable and chemically inert molecule with the C=O bond possessing a high dissociation energy of ~750 kJ/mol and C-C bond of ~336 kJ/mol, indicating a significant amount of energy input [16,17]. Moreover, the breaking of the C=O bonds and the bending of the molecular from linear to bent has been shown to require a large input of energy [18]. In addition, Equation (9) has been shown to be the necessary step to activate CO_2_, which has a very negative potential; therefore, high overpotential is needed to convert a CO_2_ molecule to products [9,13].

CO_2_ + e^−^ → CO_2_^•−^   E° = −1.90 V vs. NHE (9)

We chose to highlight bicarbonate reduction in this study because of its reduction efficiency over dissolved CO_2_ gas due to its limited solubility of CO_2_ in water (0.033 M at 298 K and 1 atm) [19]. As several reports require the use of aqueous conditions, bubbled CO_2_ poses a concentration disadvantage. In a previous study by our group [20], we questioned the standard assumption that CO_2_ was the only species undergoing reduction even though numerous publications were using a bicarbonate buffer. This paper was among the first to conduct a control experiment with only bicarbonate buffer and no bubbled CO_2_ [21,22]. From this control experiment, we discovered that bubbling CO_2_ did not result in significantly higher formate production. Consequently, we decided to focus our efforts on bicarbonate reduction due to the following major issues with CO_2_. First, a considerable practical problem with atmospheric capture is that the concentration of CO_2_ in the air is still fairly low at 100’s of ppm [23]. While several concentrated CO_2_ sources exist at fossil fuel burning plants or in some cases oil drilling activities, it is very hard to supply a reactor with enough CO_2_ if it is being drawn from the atmosphere. Our experimental conditions parallel ocean bicarbonate concentration of 0.033 M [19] and with the continual acidification of the ocean due to dissolved carbon dioxide, it is relevant to capture bicarbonate and convert it to a value-added product.

## 3. The Case for Photons to Formate

In comparison to hydrogen, fuels based on carbon hold the advantage when it comes to denser volumetric energy along with transportation and storage within the current industrialized infrastructure. Converting CO_2_ into value-added chemicals could contribute to negative emission technologies (NETs) by carbon sequestration into acetic acid, ethylene and formic acid instead of expensive geological storage [11]. Of these commodity chemicals, formate production and its growing chemical application has increased significantly over the past decade [24]. Furthermore, in relation to higher C_n_ homologs, CO_2_ reduction to specialty chemicals in the range of C_1_-C_3_ such as formic acid production (0.2 MtC/yr) or propanol (0.1 MtC/yr) offers a brighter outlook for near term economic sustainability [25,26,27]. By comparison, carbon mitigation at the GtC/yr scale, which is ultimately impactful toward global climate change, will necessitate chemical conversion of greenhouse gases to be competitive with the production of fossil fuels such as coal (4 GtC/yr) and natural gas (1.4 GtC/yr) [28,29].

### Techno-Economic Analysis

Even though CO_2_ reduction is a widely studied field, much of the research is focused on the fundamental aspects of catalyst design and product selectivity. However, for this process to be commercialized, many requirements need to be met such as (1) continuous operation, (2) high product selectivity and throughput and (3) long-term stable operation [30]. In addition, it is essential that the energy used in the CO_2_ conversion process does not generate additional CO_2_. Recent studies on the economic feasibility of a commercial CO_2_ reduction reactor show that formic acid is one of the only products that has potential to be made on a large-scale basis. Jouny et al. calculated the end-of-life net present value (NPV) of a CO_2_ electrolyzer for the production of 100 tons/day of various CO_2_ reduction products and found that only carbon monoxide and formic acid were the only economically viable products with NPVs of $13.5 million and $39.4 million, respectively [25]. Spurgeon and Kumar analyzed electrochemical conversion of flue gas CO_2_ to liquid products and found that electrosynthesized formic acid from CO_2_ was analyzed to be near market prices as a bulk chemical, even though it is not promising as an economic fuel. The major issue was the high capital cost for CO_2_ electrolyzers since high Faradaic efficiency, high current densities and reduced electrolyzer cost is necessary for economic viability [31]. Rumayor et al. carried out a techno-economic analysis of electrochemical reduction of CO_2_ to formic acid and found that it is not yet profitable and competitive under current market conditions due to the high production costs [32].

## 4. Photocatalytic CO_2_ Reduction to Formic Acid

### 4.1. Semiconductors

Halmann and Inoue were the first to reduce CO_2_ with semiconductor photocatalysts. Within a year of each other, both published their works in *Nature* reported the photo-reduction of CO_2_ in aqueous medium. In 1978, Halmann constructed a photoelectrode consisting of p-type gallium phosphide that produced formic acid after 18 h of irradiation [33]. A year later, Inoue reported the reduction of CO_2_ using several photosensitive semiconductor powders (TiO_2_, ZnO, CdS, GaP, SiC and WO_3_) suspended in water [34]. Inoue’s work was one of the first studies to hypothesize that for photocatalysis to be efficient, photo-excited electrons in the more negative conduction band have greater ability to reduce carbon dioxide. Even though this work produced low quantum yields (0.0005%), it was an important early study that inspired many other researchers. Following these two papers, both authors continued to make novel discoveries in this field. Halmann, in 1982, demonstrated enhanced formate production could be achieved by using oxides of rare earth dopants (Eu_2_O_3_, Sm_2_O_3_, Nd_2_O_3_, CeO_2_) to dope large band gap semiconductors (BaTiO_3_, LiNbO_3_) [35]. The optical properties of the catalysts were studied by diffuse reflectance spectroscopy, which uncovered the fact that rare earth dopants allowed the extension of optical absorption in the visible region of approximately 600 nm. A year later, Halmann reported the photoelectrochemical reduction of aqueous CO_2_ using a single crystal p-gallium phosphide and p-gallium arsenide as photocathodes in neutral or weakly acidic medium [36]. The main product was determined to be formate (71 µmol) but formaldehyde and methanol were also produced. The influence of pH on the behavior of the electrodes was studied and a strong photo-effect was seen for all pH values. The next year, Halmann once again studied TiO_2_ in the photo-reduction of CO_2_, this time doped with RuO_2_ [37]. They found that by doping anatase TiO_2_ with RuO_2_, formate was the predominant product and efficiency reached 0.04%. Later in 1995, Inoue reported on the photoreduction of CO_2_ using chalcogenide semiconductor microcrystals. This study synthesized cadmium-loaded ZnS microcrystals in a sodium bicarbonate buffer and bubbled CO_2_, which produced the highest quantum efficiency of 32.5%, twice as large as that obtained on bare ZnS microcrystals. However, the quantum efficiency was determined using 280 nm irradiation, which would explain the high efficiency by using monochromatic light. 2-propanol was used as a hole scavenger and it was found that Cd loading was an important factor in formate production. It was also reported that with increasing mole fraction of Cd, formate production actually decreased and that the optimal Cd loading is 0.5–0.67%. Other metals such as Pb, Ni, Ag and Cu were used but none produced as high efficiency as Cd-ZnS [38].

Since the work by Inoue, ZnS has become an attractive semiconductor photocatalyst because of its non-toxic and earth abundant attributes and its effectiveness in the reduction of CO_2_. Kanemoto was able to achieve sufficient formate production of 75.1 µmol/h but this experiment was performed at 313 nm irradiation [39]. These findings also confirmed 2-propanol to be the most efficient electron donor amid sodium phosphate, sodium sulfate and triethanolamine. Johne and Kisch loaded ZnS onto large surface area SiO_2_ particles to produce formate under UV irradiation. Later, 2,5-dihydrofuran was used as a reducing agent, which assisted in the production of 7000 µmol of formate [40].

**Figure 3:**
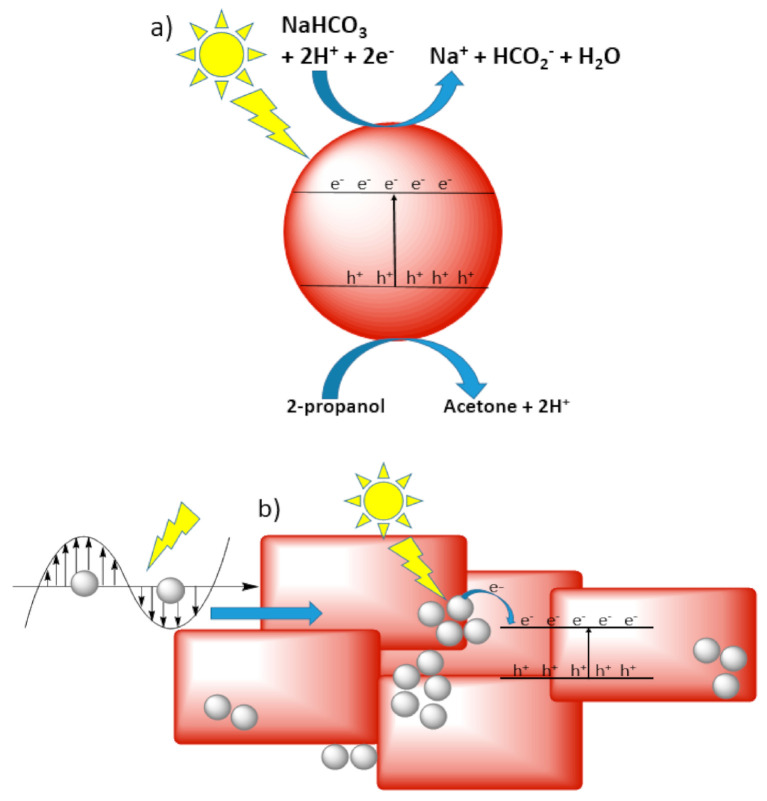
Proposed mechanism for (**a**) Cu_2_O semiconductor and (**b**) Ag/Cu_2_O (blue block arrow represents resonant energy transfer from metal to semiconductor). Reproduced from [41], with permission from American Chemical Society, 2018.

Our group recently explored the photoreduction of bicarbonate to formate using wurtzite and sphalerite ZnS particles [20]. ZnS was studied in depth under the parameters of size, crystal lattice, surface area and band gap on the productivity of formate. Photochemical experiments were performed under air mass coefficient (AM 1.5 and 0) solar simulator conditions. Formate production was negligible under AM 1.5 conditions but significantly increased under AM 0 conditions due to the inclusion of shorter wavelength photons. This work was one of the first to question the standard assumption that CO_2_ is the only species undergoing reduction. Compared to earlier works reporting bubbled CO_2_ in the presence of bicarbonate, this work suggests that bicarbonate was the predominant species undergoing reduction. As the C-H bond undergoes oxidation in 2-propanol, glycerol with three hydroxylic positive hole scavengers was hypothesized as an improved semiconductor hole-scavenger and green chemistry solvent glycerol greatly improved the efficiency of the reaction. Continuing with ZnS, our group studied copper (I) oxide (Cu_2_O) in the photoreduction of bicarbonate to formate. This work examined micron- and nano-particulate Cu_2_O and synthesized a Ag/Cu_2_O nanocomposite [41]. Due to plasmon-induced electron transfer from silver to Cu_2_O, an enhanced formate production was found with the Ag/Cu_2_O nanocomposite photocatalyst at 5.5% apparent quantum efficiency (Figure 3). In addition, an Au/TiO_2_ and Ag/TiO_2_ nanocomposite photocatalyst could reach apparent quantum efficiencies of 2.4% and 7.8%, respectively [42,43]. Beierle et al. designed a titanium nitride-titanium dioxide nanocomposite for bicarbonate reduction to formate. This work demonstrated that under solar illumination, the TiN-TiO_2_ nanocomposite has higher photocatalytic activity than bare TiN or TiO_2_, this nanocomposite can also greatly enhance formate production and remain stable for 8 h [44].

Baran et al. synthesized nanocrystalline zinc sulfide surface-modified with ruthenium(0) for the photocatalytic CO_2_ reduction to formate, carbon monoxide and methane [45]. Formation of acetone was also observed due to the oxidation of the hole scavenger isopropanol. Surface-modified ZnS was more efficient than pure ZnS due to (1) the lowering of the CO_2_ activation energy, (2) collection of electrons at Ru particles resulted in a better charge separation and (3) better CO_2_ desorption at the surface of modified ZnS. Both polar and non-polar solvents were tested and it was reported that in a polar solvent (water), formate is the major product and water was a better H-transfer agent than isopropanol. Kuwabata et al. employed ZnS microcrystallites for photocatalytic CO_2_ reduction to methanol and formate by using methanol dehydrogenase and (MDH) and pyrroloquinoline quinone (PQQ) as an electron mediator for MDH and 2-propanol as a positive hole scavenger [46]. Acetone production resulting from the oxidation of 2-propanol was also studied.

Irvine et al. used CdS, ZnO, SiC, BaTiO_3_ and SrTiO_3_ to photochemically reduce aqueous CO_2_ [47]. Product analysis showed the formation of formate, formaldehyde and methanol; the highest efficiency was obtained with ZnO, CdS and SiC. Continuing on Irvine’s work, Eggins et al. used CdS or ZnS colloids for the photo-reduction of CO_2_ to produce formate and other dimeric and tetrameric products such as oxalate, glyoxylate, glycolate and tartrate. Other semiconductors studied included ZnO, SiC, BaTiO_3_ and SrTiO_3_ [48]. This work discusses the role of several hole acceptor (or electron donor) compounds that assist in reacting with photogenerated holes. Different electron donors tested include iron ferracyanide, hydroquinone, 2-propanol, RuO_2_ and hypophosphite. This paper highlights that semiconductors with the most negative conduction band potential results in the best quantum yields because they have a stronger reducing potential. This work concluded that ZnS resulted in the highest quantum yield among all the semiconductors tested. Henglein et al. reported the efficient photoreduction of CO_2_ to formic acid using SiO_2_-stabilized ZnS and 2-propanol as electron donor. By employing this system 0.80% AQE was achieved [49].

**Figure 4:**
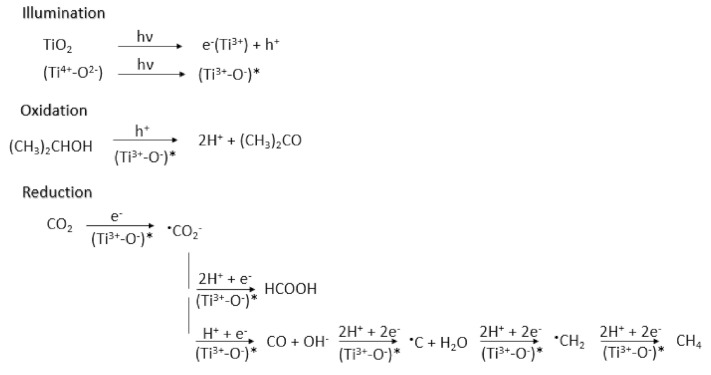
Proposed mechanism of photocatalytic reduction of high-pressure CO_2_ using TiO_2_ and 2-propanol as a hole scavenger. Reproduced from [51], with permission from Elsevier, 1998.

Kaneco and coworkers published three studies, all employing TiO_2_ in a liquid CO_2_ medium [50,51,52]. In the first effort, the effect of temperature, pressure, illumination time and surface area were examined. It was found that the formate yield was neither affected by temperature nor pressure. Formate production increased sharply with illumination time up until 5 h and progressed slowly until 30 h. A similar trend was observed with the surface area of the catalyst, formate increased linearly with increasing surface area of TiO_2_ up to 0.4 m^2^/g. Electron spin resonance elucidated the reaction mechanism of the reaction, which identified two radical species, photogenerated Ti^3+^ and CO_2_^•−^ radical anion which resulted from the first electron transfer to CO_2_. In their follow-up work, Kaneco and coworkers continued to investigate the photoreduction of high-pressure CO_2_ using TiO_2_ powders with 2-propanol as a positive hole scavenger [51]. Methane was found to be the main reduction product; formate was also detected but only under extremely high pressures of CO_2_. In 1999, Kaneco and coworkers continued to study TiO_2_ powders in supercritical fluid CO_2_ by the investigation on the effects of temperature, pressure, irradiation time, surface area and aqueous solution for protonation. This study determined that formate production increased with an acidic pH medium because H^+^ are advantageous for desorption of reaction intermediates from the surface of TiO_2_ [52]. These two works were the first to point out that photo-excited TiO_2_ results in (Ti^3+^-O^−^)* species (Figure 4) [51,52].

Liu et al. studied photocatalytic CO_2_ reduction on CdS particles with and without surface modification in various solvents [53]. Formate and carbon monoxide were the two major products, as well as acetone from the oxidation of 2-propanol. The type of solvent and surface modification played a major role in the selectivity of the products. The reduction of CO_2_ in acetonitrile produced carbon monoxide and formate, while in dichloromethane produced carbon monoxide only. Kisch reported CdS supported on silica and zinc sulfide to photocatalyze the reduction of bicarbonate to formate [54]. For CdS supported on zinc sulfide, it was found that the optimal CdS loading was 5%, which increased formate production 40-fold and 16-fold as compared to bare CdS and ZnS. Formate production was found to be dependent on bicarbonate concentration, when bicarbonate concentration increased from 33 to 60 mM formate production was enhanced by 10%. Formate production decreased by 20% when the pH was lowered to 5.3 but at a pH of 11.2, formate production increased by 25%. Zhang et al. used manganese sulfide (MnS) to reduce bicarbonate to formate at neutral pH [55]. Both ZnS and MnS have their conduction bands at highly reducing positions at −1.04 and −1.19 V versus NHE. This work results in quantum efficiency of 4.2%, however, the wavelengths utilized fell within the UV region of the solar spectrum, between 200 and 400 nm.

**Figure 5:**
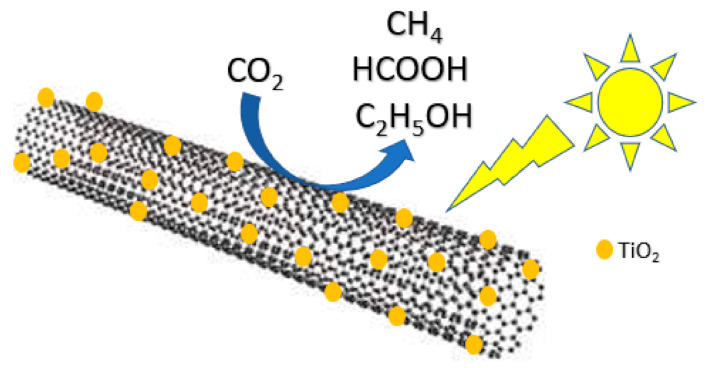
Photocatalytic CO_2_ reduction to CH_4_, HCOOH and C_2_H_5_OH using TiO_2_-decorated multi-walled carbon nanotube (MWCNT).

**Figure 6:**
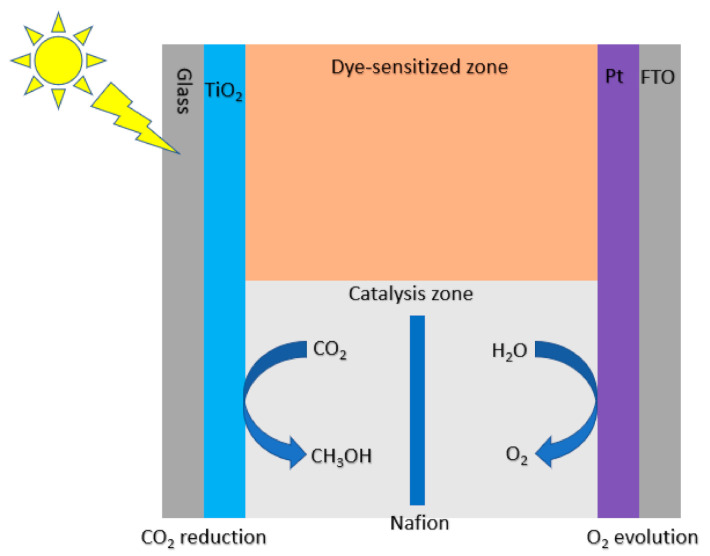
Proposed mechanism for the photocatalytic CO_2_ reduction using bifunctionalized TiO_2_ film.

Xia et al. synthesized a multi-walled carbon nanotube (MWCNT) supported TiO_2_ and found that the addition of MWCNT greatly enhanced the photocatalytic activity of TiO_2_ (Figure 5) [56]. As with other doped catalysts, it was found that there is an optimal amount of MWCNT before it begins to decrease the photocatalytic activity of TiO_2_ due to the fact that excessive MWCNT can shield the TiO_2_ from absorbing UV light. The optimal amount of MWCNT prevents TiO_2_ from aggregating and can delay electron/hole recombination. Qin et al. made a bifunctionalized TiO_2_ film containing a dye-sensitized zone and a catalysis zone designed for visible light photocatalytic CO_2_ reduction (Figure 6) [57]. Electrons transferred from the dye to the conduction band of TiO_2_ resulted in highly efficient conversion of CO_2_ to formate, formaldehyde and methanol. After the electron injection, the dye is oxidized to dye^+^, which can be regenerated by accepting electrons from I^−^ from the electrolyte. Ulagappan et al. studied the photoreduction of gaseous CO_2_ in Ti-silicate molecular sieves using methanol as an electron donor [58]. The main result of the study is the detection of formate and it was proposed that upon excitation of the metal center, transient Ti^+III^ reduces CO_2_ while methanol is oxidized.

**Figure 7:**
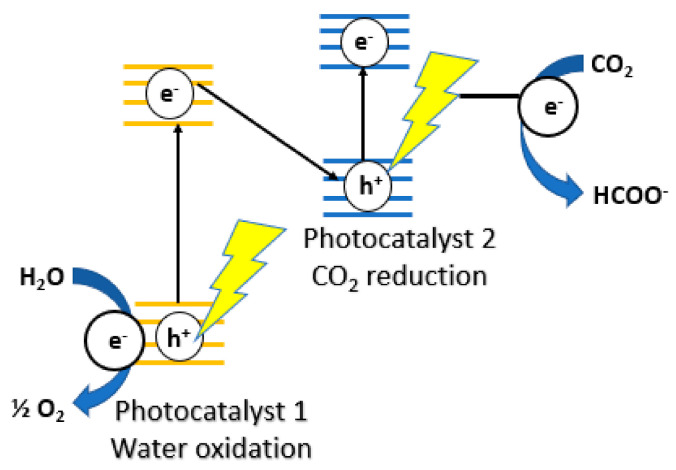
Illustration of the Z-scheme for CO_2_ reduction. Reproduced from [60], with permission from American Chemical Society, 2011.

In the 2000s, doping, co-catalyst and extending optical absorption to the visible region became a popular area for research. Sato et al. coupled a p-type semiconductor (N-doped Ta_2_O_5_) with a ruthenium complex as an electrocatalyst to produce formate from CO_2_ [59]. This was the first report of electron transfer from semiconductor in the excited state to a metal complex in the ground state. Triethanolamine (TEOA) was used as an electron donor as well as a proton source and the measured quantum yield was 1.9% at 405 nm. A year later, Sato et al. studied the photoelectrochemical reduction of CO_2_ over p-type InP/Ru complex polymer hybrid photocatalyst [60]. This reduction, using H_2_O as an electron donor and proton source was achieved by using a two-step excitation process called a Z-scheme (Figure 7). Highly selective CO_2_ photo-reduction was achieved by Arai and coworkers by combining Cu_2_ZnSnS_4_ (CZTS) with a metal complex electrocatalyst [61]. The main product was identified to be formate, which demonstrates the selectivity and efficiency of the hybrid semiconductor-metal-complex-electrocatalyst catalyst. Suzuki et al. continued on that idea of p-type photoactive N-doped Ti_2_O_5_ with a ruthenium complex, which can efficiently reduce CO_2_ to formate using visible light [62].

Co-catalysts became a popular method to enhance semiconductor efficiency. Iizuka et al. used Ag as a co-catalyst for ALa_4_Ti_4_O_15_ (where A = Ca, Sr and Ba) to reduce CO_2_ to formate [63]. No sacrificial agent was used because water was consumed as a reducing agent (electron donor). It was found that the optimum Ag loading was 2 wt% and that BaLa_4_Ti_4_O_15_ was the most active photocatalyst. Palladium is another noble metal that is often used as a co-catalyst. Raja et al. synthesized two photocatalysts-anodized titanium oxide nanotubes (T-NT) and bismuth titanate (BTO) decorated with palladium nanoparticles [64]. Pd-BTO showed a 2-fold increase in formic acid (110 µmol/h/g) yield when compared to bare BTO or T-NT, which is attributed to better charge separation in the hybrid Pd-BTO and the higher visible light harvesting ability of BTO because of its lower bandgap.

Mendoza et al. employed Co_3_O_4_ powders for CO_2_ photo-reduction under visible light irradiation [65]. Co_3_O_4_ is a p-type semiconductor with band gap of 2.0 eV and has a conduction band edge that is more negative than the potentials of the CO_2_ reduction reactions. In this study, formate was produced without any hole scavenger nor photo-sensitizer. Qin et al. synthesized a bismuth yttrium oxide (BiYO_3_) photocatalyst for CO_2_ reduction to formate under visible light irradiation [66]. Due to its large surface area and smaller band gap, BiYO_3_ produced fewer hydroxyl radicals to give a higher formate yield.

Most recently, copper oxide and copper oxide-derivatives have been gaining attention as a highly active and earth abundant photocatalyst. Ali and coworkers designed a Z-scheme heterostructure composed of reduced titania and Cu_2_O capable of reducing CO_2_ to CH_4_. Due to the Z-scheme architecture, Cu_2_O was protected against photocorrosion and the photocatalyst was stable for 42 h. In addition, the synergistic interactions between reduced titania and Cu_2_O resulted in 0.13% photoreduction of CO_2_ to CH_4_ [67]. Yin et al. grafted copper oxide nanoclusters onto niobate nanosheets, which served as a light harvesting component. This work found that photogenerated holes in the valence band of niobate nanosheets react with water and photogenerated electrons in the conduction band is injected into the copper oxide nanoclusters resulting in the production of carbon monoxide [68]. Lan et al. loaded Cu nanoparticles onto TiO_2_ which exhibited significantly higher photocatalytic activity than pure TiO_2_ for CO_2_ reduction and selective for carbon monoxide [69]. Bae et al. synthesized zinc oxide-copper (I) oxide hybrid nanoparticles in colloidal forms with copper (I) oxide nanocubes bound to zinc oxide. This photocatalyst exhibited high selectivity for methane (>99%) which was attributed to the optimal band alignment of zinc oxide and copper (I) oxide, surface defects, high surface area and colloidal morphology of the catalyst [70]. Nogueira et al. evaluated the effect of electrolyte on photochemical CO_2_ reduction using CuO nanoparticles and found electrolyte strongly influences product selectivity. NaOH led to methane production while Na_2_C_2_O_4_ led to carbon monoxide production and KBrO_3_ led to oxygen production [71]. Dedong et al. synthesized a Cu_2_O/coal-based carbon nanoparticle hybrid to reduce CO_2_ to methanol [72]. Zhang et al. fabricated an Ag-Cu_2_O/ZnO nanorod hybrid catalyst and found Cu_2_O enhances the CO_2_ chemisorption on the catalyst surface while the formation of Z-scheme system between Cu_2_O and ZnO facilitates charge separation. Silver nanoparticles onto Cu_2_O leads to higher photocatalytic activity due to electron transfer from silver to Cu_2_O [73]. Aguirre et al. found that TiO_2_ can protect Cu_2_O from undergoing photocorrosion when a Cu_2_O/TiO_2_ hybrid catalyst is formed. This hybrid creates an efficient Z-scheme which facilitates electron transfer from TiO_2_ to Cu_2_O [74]. Park et al. synthesized both CuO-TiO_2_ and Cu_2_O-TiO_2_ and demonstrated this hybrid catalyst has enhanced light absorption and rapid charge separation due to the intrinsic p-n heterojunction of the material, leading to improved photocatalytic activity [75]. Gusain et al. prepared reduced graphene oxide-copper oxide nanocomposites by covalent grafting of CuO nanorods onto reduced graphene oxide. Bare CuO showed low photocatalytic activity due to rapid charge carrier recombination but rGO-Cu_2_O and rGO-CuO showed significantly higher photocatalytic activity in the reduction of CO_2_ to methanol under visible light irradiation [76]. Zhai et al. synthesized a core-shell structure containing two co-catalysts capable of reducing CO_2_ to methane and carbon monoxide. They propose that the Cu_2_O shell provides sites for CO_2_ reduction while the Pt core extracts photogenerated electrons from TiO_2_ [77]. Wu et al. observed that the (110) facet of a single Cu_2_O particle is capable of reducing CO_2_ to methanol while the (100) facet is inert. The oxidation state of the active sites changes from Cu(I) to Cu(II) due to CO_2_ and H_2_O adsorption and changes back to Cu(I) after CO_2_ conversion under visible light irradiation [78]. Table 2 summarizes the different types of semiconductors used in photocatalytic CO_2_/bicarbonate reduction to formate and other products.

### 4.2. Phthalocyanine-Semiconductor Composites

Phthalocyanines are macrocyclic heterostructures with aromatic conjugated complexes consisting of nitrogen and carbon atoms. They offer enormous potential as the light harvesting components of dye-sensitized semiconductors [79]. They also offer high photo and thermal stability and low toxicity. In addition, the close position of the TiO_2_ conduction band with respect to the LUMO orbital energy of the phthalocyanines favors efficient charge transfer. When the phthalocyanines molecule is excited by visible light, electrons are excited from the HOMO to the LUMO energy level and is then injected into the conduction band of TiO_2_.

**Figure 8:**
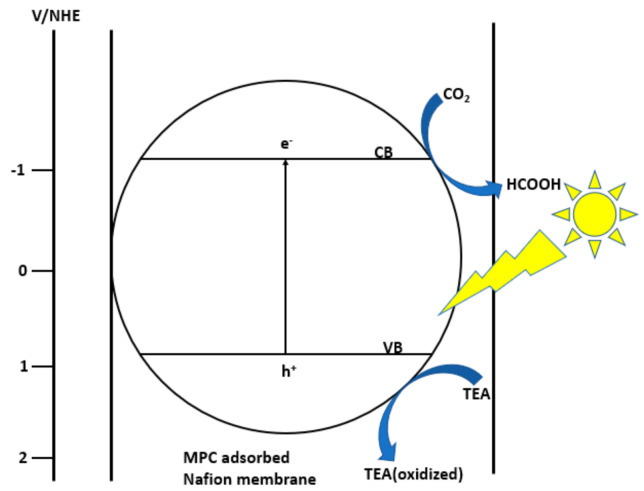
Schematic illustration of the photocatalytic CO_2_ reduction at MPC adsorbed in a Nafion membrane. (Circle represents the semiconducting nature of the adsorbed MPC Nafion membrane. MPC = CoPC or ZnPC, TEA = triethanolamine, CB = conduction band, VB = valence band and EF = Fermi level).

**Figure 9:**
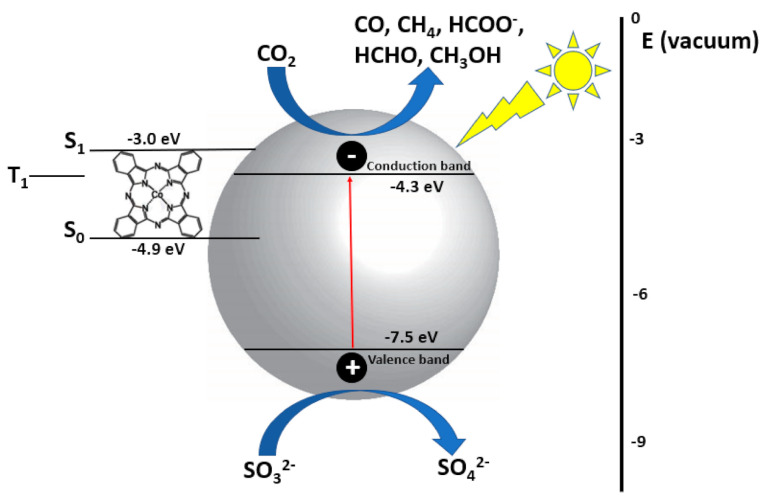
Photocatalytic CO_2_ reduction using CoPc-loaded TiO_2_.

In 1997, Premkumar et al. was able to produce formate from carbon dioxide upon photo-reduction by metal phthalocyanines (cobalt and zinc) adsorbed onto a Nafion membrane that acted as a photocatalyst in acidic aqueous solution containing triethanolamine as a hole scavenger [80,81]. The catalyst was immobilized in the Nafion membrane and because they are physically separated from the solution phase they are able to exhibit high photocatalytic efficiency (Figure 8). It was found that the membranes behave as a p-type semiconductor and high turnover numbers were achieved. In 2009, Liu et al. synthesized CoPc-loaded TiO_2_ and in the presence of NaOH, produced high formate yield [82]. Na_2_SO_3_ was used as a hole scavenger and under optimal conditions, 1032 µmol/g cat of formate was produced. It was found that with the addition of CoPc, formate production significantly increased, with optimal CoPc loading being 0.5 wt%. The contact of TiO_2_ and CoPc involves redistribution of charge. Because the oxidation potential of S_1_ of CoPc is higher than the conduction band of TiO_2_, while the energy of T_1_ is lower than the conduction band of TiO_2_ (Figure 9), it is thermodynamically feasible for electron transfer from CoPc to TiO_2_. When this occurs, CoPc is oxidized to CoPc^*+^. The influence of NaOH, hole scavenger and irradiation time has been investigated. Because NaOH can dissolve more CO_2_ than pure water, making HCO_3_^−^ the predominant form in aqueous solution therefore accelerating the photoreduction [82]. The mechanism of CO_2_ reduction with CoPc-loaded TiO_2_ begins with photo-excitation of the CoPc from S_0_ to S_1_, which can subsequently transfer its electrons to the conduction band of TiO_2_. CO_2_ molecules adsorbed onto the surface of TiO_2_ gain electrons and are reduced (Equations (10) and (11)) [81].

CoPc (S_0_) + hυ → CoPc*(S_1_) (10)

CoPc*(S_1_) + TiO_2_(CB) → CoPc^•+^ + e^−^(TiO_2_/CB). (11)

In 2009, Zhao et al. also synthesized a CoPc/TiO_2_ nanocomposite to reduce CO_2_ under visible light irradiation [83]. CoPc molecules are excited first and are able to inject their electrons into TiO_2_, this allows for the increased separation of electron/hole pairs and thus increasing the photocatalytic efficiency to 1714 µmol/g cat. In 2013, Yazdanpour et al. synthesized copper phthalocyanine-modified TiO_2_ (CuPc/TiO_2_), which was coated on a stainless steel mesh and used for the photo-reduction of CO_2_ in the presence of visible light [84]. Mele et al. synthesized copper and zinc phthalocyanine-loaded TiO_2_ under visible light irradiation [79]. They reported that CuPc/TiO_2_ was the most efficient yielding formate production of 208.5 µmol/g cat. In 2015, Mele expanded on their work of CuPc-loaded TiO_2_ by studying both Cu(II) porphyrin and Cu(II) phthalocyanine loaded onto TiO_2_ [85]. Cu(II) phthalocyanine-loaded TiO_2_ was more efficient in the photoreduction of CO_2_ to formate due to its favorable reduction potential. This work reported a detailed study of the effect of the amount of catalyst, initial pH, copper loading in the CuPc-TiO_2_ composite, irradiation source and sensitizers. This work reported production values for formate of 239.5 µmol/g cat. Table 3 summarizes various phthalocyanine-enhanced semiconductors to reduce CO_2_/bicarbonate to formate and other products. 

### 4.3. Metal-Organic Frameworks

Metal-organic frameworks (MOFs) are a class of crystalline materials made by joining metal ions or clusters and organic linkers together. Inorganic metals are called secondary building units (SBU) and organic units are ditopic or polytopic organic carboxylates. The different combinations of organic and inorganic components, as well as varying the geometry, size and functionality has led to more than 20,000 different MOFs being synthesized [86]. The three most distinguishing features of MOFs are their large surface area and ultrahigh porosity; as well as its permanent porosity. MOFs are stable structures that do not collapse upon the removal of solvent. Compared to commercial TiO_2_ (P-25) that have surface area of only 35–65 m^2^/g, MOFs have a tremendous amount of surface area. Surface area of MOFs typically range from 1000 to 10,000 m^2^/g and their porosity is greater than 50% of the MOF crystal volume [86]. MOFs are also shown to be thermally and chemically stable. It is these properties of MOFs that give them a great advantage over other porous materials such as zeolites and carbon-based materials.

Metal-organic frameworks (MOFs) are emerging as a new type of promising photocatalysts. MOFs are a class of crystalline and microporous material with a vast array of topologies and applications in numerous fields such as gas sensing, catalysis, gas storage and separation and drug delivery [87]. In MOFs the metal clusters can be regarded as inorganic semiconductor quantum dots that are linked by organic linkers serving as antennas to activate the quantum dots [88]. Electron injections occurs from the photo-excited organic linkers to the metal clusters, termed linker-to-cluster charge transfer (LCCT). MOFs are superior to semiconductors because their light absorption ability can be easily tuned by modifications on the organic linkers to make them visible-light responsive [89]. Table 4 summarizes CO_2_ reduction using various metal-organic frameworks. 

**Figure 10:**
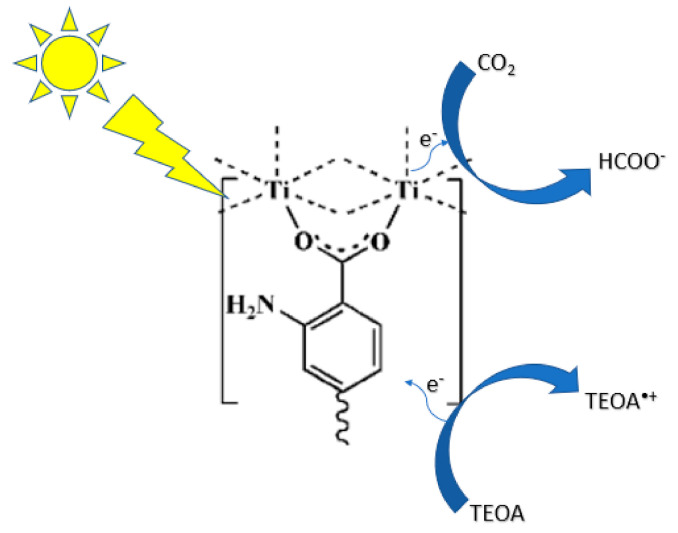
Proposed mechanism for the photocatalytic CO_2_ reduction using NH_2_-MIL-125(Ti) under visible light irradiation.

**Figure 11:**
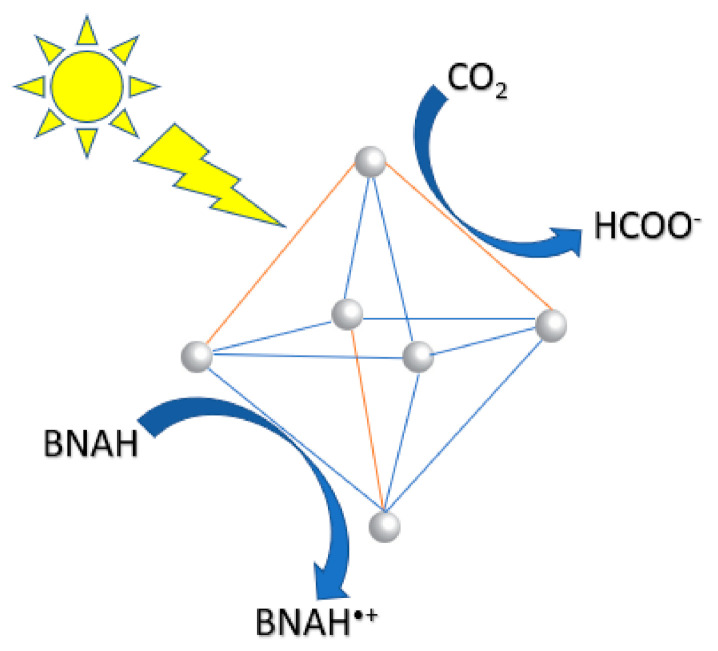
Proposed mechanism for photocatalytic formate production using UiO-67-Mn(bpy)(CO)_3_Br.

Fu et al. synthesized a Ti-based MOF Ti_8_O_8_(OH)_4_(BDC)_6_ (where BCD = benzene-1,4-dicarboxylate) and the photocatalytic reduction of CO_2_ was performed in acetonitrile with triethanolamine (TEOA) as a sacrificial agent under visible light irradiation [90]. This MOF exhibited extremely high surface area of 1302 m^2^/g that produced 8.14 µmol of formate after 10 h. Upon irradiation in the LMCT band, a long-lived excited charge separate state occurs by transferring an electron from the organic ligand to Ti^4+^ (Figure 10). Sun et al. synthesized NH_2_-UiO-66(Zr), which is a Zr-containing MOF composed of hexameric Zr_6_O_32_ units linked by benzenedicarboxylate (BDC) [88]. This study substituted the BDC ligand with 2-aminoterephthalic acid (ATA), which rendered the MOF visible-light responsive due to the amino substituent. In the presence of triethanolamine as a sacrificial agent, visible light irradiation was able to reduce CO_2_ to formate reaching production values of 20.7 µmol/g cat-hr. Wang et al. reported synthesizing a series of earth-abundant Fe-containing MOFs that are able to reduce CO_2_ under visible light irradiation [89]. The direct excitation of the Fe-O clusters induces the electron transfer from O^2−^ to Fe^3+^ to form Fe^2+^, which is responsible for the CO_2_ reduction. In addition, when these MOFs are functionalized with an amine substituent, their catalytic efficiency is greatly increased. This is attributed to the existence of dual excitation pathways: excitation of the NH_2_ functionality is followed by electron transfer to the Fe center and the direct excitation of Fe-O clusters. In the presence of TEOA as a sacrificial agent, formate production reached 178 µmol. Fei et al. incorporated a manganese bipyridine complex [Mn(bpydc)-(CO)_3_Br] into a robust Zr(IV)-based MOF for CO_2_ reduction to formate [91]. [Ru(dmb)_3_]^2+^ was used as a photosensitizer and 1-benzyl-1,4-dihydronicotinamide (BNAH) was used as a sacrificial agent (Figure 11). The enhanced photocatalytic efficiency was ascribed to the structure of the framework providing isolated active sites, which stabilize the catalyst. The catalyst maintained its crystallinity and was reused over several runs. Most recently, Lee et al. synthesized a mixed metal (Zr/Ti) MOF to reduce CO_2_ to formate under visible light irradiation [92]. TEOA was used as a sacrificial base and 1-benzyl-1,4-dihydronicotinamide (BNAH) as a sacrificial reductant. The MOFs were studied by photoluminescence spectroscopy to study charge transfer from organic linker to the metal cluster. This work resulted in high turnover numbers and the catalysts were recyclable over three runs.

## 5. Future Perspectives and Outlook

The majority of this review is focused on semiconductors for CO_2_/bicarbonate reduction because they are the most widely used photocatalyst. However, quantum efficiencies are still low due to extremely rapid electron/hole recombination within the semiconductor, narrow optical absorption of the semiconductor, scattering of the semiconductor, competing hydrogen evolution reaction and products getting re-oxidized to CO_2_. Though these issues can be mitigated by using photosensitizers and hole scavengers, a lot of work still needs to be done to make the reaction more efficient. In addition, semiconductors eventually degrade over time, so more durable catalysts need to be designed. Plasmon-enhanced photocatalysis is a rapidly expanding field due to the excellent optical properties of plasmonic noble-metal nanoparticles such as Au and Ag. Plasmonic nanoparticles primarily absorb in the visible region of the solar spectrum and are known to generate hot electrons that can be injected into the conduction band of semiconductors, allowing for excess electrons available for reduction. Plasmonic nanoparticles, along with organic dyes, can act as excellent photosensitizers for semiconductors.

Moving forward, design strategies for photocatalysts include the fabrication of three-dimensional hierarchical nanostructures to maximize surface area, catalyst kinks and defects and grain boundaries to allow for molecular adsorption to the catalyst surface. Different metal crystal structures and surface facets lead to different product distribution, therefore tuning the metal surface structure will allow for precise control over product selectivity. The addition of photosensitizers to render a catalyst visible light-responsive will maximize the region of optical absorption and the ability to utilize more of the solar spectrum. With these techniques, rational design of efficient, selective and durable catalysts is possible. With the development of these catalysts, one can then scale up and design reactors that can convert CO_2_ or flue gas into value-added products.

## 6. Conclusions

In conclusion, many significant achievements have been made in the field of photocatalytic CO_2_ reduction. In relation to hydrogen production, which remains an impactful renewable energy source, the case is made for the chemical conversion of CO_2_ to formate in terms of near term economic feasibility and in juxtaposition to geological storage and sequestration. Based on this review, one can see that the majority of photocatalysts for CO_2_ reduction to formate are comprised of semiconductors. However, due to their narrow region of optical absorption, many studies have incorporated ruthenium complexes, phthalocyanines, and employed metal-organic framework catalysts designed to be visible-light responsive. Other catalysts include the use of graphene and graphene oxide as electron sinks to improve charge separation. Albeit much effort has been put into studying CO_2_ reduction, the photoconversion efficiency and selectivity for desired products is still low. The mechanism of CO_2_ reduction still needs to be investigated in depth. The one-electron induced activation of CO_2_ into the anion radical CO_2_^•−^ still requires a high overpotential, which seems to be the rate-limiting step. The development of photocatalytic materials for CO_2_ reduction is rapidly expanding and will continue to reach higher efficiencies. As apparent quantum efficiencies improve with current research trends and new investigators entering the field, we assert that more practical device and design will become a major research component to the success of greenhouse gas mitigation and regenerative energy. 

## Figures and Tables

**Table 1:** The main products of CO_2_ and water reduction and the corresponding reduction potentials with reference to NHE at pH 7 in aqueous solution [13,15].

Product	Reaction	E° (V vs. NHE)	Equation
Hydrogen	2H_2_O + 2e^−^ → 2OH^−^ + H_2_	−0.41	(1)
Methane	CO_2_ + 8H^+^ + 8e^−^ → CH_4_ + 2H_2_O	−0.24	(2)
Carbon monoxide	CO_2_ + 2H^+^ + 2e^−^ → CO + H_2_O	−0.51	(3)
Methanol	CO_2_ + 6H^+^ + 6e^−^ → CH_3_OH + H_2_O	−0.39	(4)
Formic acid	CO_2_ + 2H^+^ + 2e^−^ → HCOOH	−0.58	(5)
Ethane	2CO_2_ + 14H^+^ + 14e^−^ → C_2_H_6_ + 4H_2_O	−0.27	(6)
Ethanol	2CO_2_ + 12H^+^ + 12e^−^ → C_2_H_5_OH + 3H_2_O	−0.33	(7)
Oxalate	2CO_2_ + 2H^+^ + 2e^−^ → H_2_C_2_O_4_	−0.87	(8)

**Table 2:** Summary of CO_2_ reduction using semiconductors.

Light Source	Reaction Medium and Electrolyte	Catalyst	Formate & Other Products	Ref.
Halogen lamp at 365 nm	Electrode consisting of single crystal GaP in 0.05 M K_2_HPO_4_/KH_2_PO_4_	GaP	HCOOH: 1.2 × 10^−2^ M HCHO: 3.2 × 10^−4^ M CH_3_OH: 1.1 × 10^−4^ M	[33]
500 W Xe lamp	Aqueous Suspension of semiconductor in water	TiO_2_, ZnO, CdS, GaP, SiC, WO_3_	TiO_2_: HCOOH: 1.8 x 10^−3^ M CH_3_OH: 14.6 × 10^−4^ M ZnO: HCOOH: 1.2 × 10^−3^ M CH_3_OH: 3.5 × 10^−4^ M CdS: HCOOH: 2.0 × 10^−3^ M CH_3_OH: 11.7 × 10^−4^ M GaP: HCOOH: 1.0 × 10^−3^ M CH_3_OH: 11.0 × 10^−4^ M SiC: HCOOH: 1.0 × 10^−3^ M CH_3_OH: 53.5 × 10^−4^ M WO_3_: HCOOH: 0 CH_3_OH: 0	[34]
High pressure Hg lamp	Aqueous suspensions	Doped BaTiO_3_, LiNbO_3_	HCOOH and HCHO	[35]
150 W Xe lamp	Electrodes in 0.5 M Na_2_CO_3_	Single crystal p-GaP, p-GaAs	p-GaP: HCOOH: 670 μmol HCHO: 13 μmol CH_3_OH: 10 μmol p-GaAs: HCOOH: 320 μmol HCHO: 5 μmol	[36]
High pressure Hg lamp	Aqueous suspension in water	RuO_2_-doped TiO_2_	HCOOH: 1.46 μmol/h HCHO: 0.18 μmol/h CH_3_OH: 0.2 μmol/h	[37]
High pressure Hg lamp	Aqueous suspension in 1.5 × 10^−3^ M NaHCO_3_	Cd-loaded ZnS	Quantum efficiency: HCOOH: 32.5% HCHO: 42.0% H_2_: 5.0%	[38]
High pressure Hg lamp	Colloidal suspension in 0.7 M NaH_2_PO_2_	ZnS	HCOOH: 75.1 μmol/h CO: 2.7 μmol/h H_2_: 86.0 μmol/h	[39]
Tungsten-halogen lamp (λ > 350 nm)	Aqueous suspension in water	ZnS-loaded SiO_2_	HCOOH: 10 mmol	[40]
1000 W Xenon arc lamp	Aqueous suspension in 0.2 M NaHCO_3_	ZnS	HCOOH: 140 mmol formate/g cat-hr	[20]
1000 W Xenon arc lamp	Aqueous suspension in 0.2 M NaHCO_3_	Cu_2_O	HCOOH: 2.78 mmol formate/g cat-hr	[41]
1000 W Xenon arc lamp	Aqueous suspension in 0.2 M NaHCO_3_	Au/TiO_2_	HCOOH: 55 mmol formate/g cat-hr	[42]
1000 W Xenon arc lamp	Aqueous suspension in 0.2 M NaHCO_3_	Ag/TiO_2_	HCOOH: 3.89 mmol formate/g cat-hr	[43]
1000 W Xenon arc lamp	Aqueous suspension in 0.2 M NaHCO_3_	TiN/TiO_2_	HCOOH: 3000 mmol formate/g cat-hr	[44]
150 W XBO arc lamp	Aqueous suspension in water	Ru nanoparticles-loaded ZnS	HCOOH:0.006 M	[45]
High pressure Hg lamp	Colloidal suspension in H_3_PO_4_	ZnS microcrystallite	HCOOH: 11.6 μmol HCHO: 11.8 μmol CH_3_OH: 1.2 μmol	[46]
Medium pressure mercury lamp	Aqueous suspension in water	CdS, ZnO, SiC, Ba TiO_3_, SrTiO_3_	HCOOH, HCHO	[47]
Medium pressure mercury lamp	Aqueous suspension in water	CdS, ZnS	ZnS: HCOOH: 320 μmol/L CdS: HCOOH: 87 μmol/L	[48]
Medium pressure mercury lamp	Aqueous suspension in water	SiO_2_-stabilized ZnS	HCOOH: 0.8% AQE	[49]
990 W Xe lamp	Aqueous suspension in water	TiO_2_	HCOOH: 8 × 10^−6^ mol/g-cat	[50]
Xe lamp	Aqueous suspension in water	TiO_2_	HCOOH: 2.3 × 10^−6^ mol/g-cat CH_4_: 1.2 × 10^−6^ mol/g-cat	[51]
990 W Xe lamp	Power in supercritical fluid CO_2_	TiO_2_	HCOOH: 9 × 10^−6^ mol/g-cat	[52]
500 W high pressure mercury arc lamp	Aqueous suspension in water and 2-propanol	CdS	HCOOH: 0.4 μmol HCHO: 1.9 μmol CO: 0.8 μmol H_2_: 0.5 μmol	[53]
100 W tungsten halogen lamp, 150 W xenon lamp	Aqueous suspension in water	CdS-SiO_2_, CdS-ZnS	CdS-SiO_2_: HCOOH: 10 × 10^−5^ M HCHO: 3.4 × 10^−5^ M CdS-ZnS: HCOOH: 4.5 × 10^−5^ M	[54]
450 W medium pressure UV Hg arc lamp	Aqueous suspension in water and 7.2 mM NaHS and 2.5 mM NaHCO_3_	MnS	HCOOH: 200 μM	[55]
15 W UV lamp λ = 365 nm	Aqueous suspension in water	MWCNT-supported TiO_2_	HCOOH: 125.1 μmol/g CH_4_: 73.33 μmol/g C_2_H_5_OH: 149.36 μmol/g	[56]
300 W Xe lamp	Electrode in water adjusted to pH 4	Dye-sensitized TiO_2_ film	HCOOH: 1.8 mmol/cm^2^ HCHO: 1.4 mmol/cm^2^ CH_3_OH: 1.9 mmol/cm^2^	[57]
Pulsed Nd:YAG laser at 10 Hz, λ = 266 nm	Methanol	Ti silicalite molecular sieve	HCOOH, CO	[58]
Xe lamp	Aqueous suspension in MeCN/TEOA	N-doped Ta_2_O_5_ (N-Ta_2_O_5_), linked with electrocatalysts [Ru(dcbpy)(bpy)(CO)_2_]^2+^ or [Ru- (dcbpy)_2_(CO)_2_]^2+^	HCOOH: 1.9% quantum yield	[59]
Solar simulator with AM 1.5 filter	Aqueous suspension in water and 10 mM NaHCO_3_	InP/Ru complex polymer hybrid	HCOOH: 4.71 μmol/cm^2^	[60]
Xe light source	Aqueous suspension in water	Cu_2_ZnSnS_4_	HCOOH: 0.22 mM	[61]
500 W Xe lamp	Aqueous suspension in acetonitrile/triethanolamine	N-doped Ta_2_O_5_ with [Ru(dcbpy)_2_(CO)_2_]^2+^	HCOOH: 1.9 quantum efficiency	[62]
400 W high-pressure mercury lamp	Aqueous suspension in water	Ag-loaded ALa_4_Ti_4_O_15_ (A = Ca, Sr, Ba)	BaLa_4_Ti_4_O_15_: HCOOH: 150 μmol	[63]
300 W solar simulator with AM 1.5 filter	Thin films submerged in 0.1 M H_2_SO_4_	Anodized titanium oxide nanotubes (T-NT), Pd-decorated bismuth titanate (BTO)	HCOOH: 160 μmol/h/g	[64]
21 W LED lamp, λ = 510 to 620 nm	Aqueous suspension in water	Co_3_O_4_	HCOOH: 4.53 µmol/g-h HCHO: 0.62 µmol/g-h	[65]
300 W Xe lamp	Aqueous suspension in 0.25 M NaOH and 0.1 M Na_2_SO_3_	BiYO_3_	HCOOH: 1.68 μmol/L	[66]
100W solar simulator with AM 1.5 filter	CO_2_ passing through solid photocatalyst	Reduced titania-Cu_2_O	CH_4_: 462 nmol/g	[67]
Xe–Hg lamp	Aqueous suspension in 0.5 M KHCO_3_	Cu(II)-grafted Nb_3_O_8_^−^ nanosheets	CO: 1.5 µmol	[68]
300 W Xe lamp	Aqueous suspension in water	Cu-TiO_2_	CO: 244 μmol/g	[69]
300 W Xe lamp	Aqueous suspension in water and 0.2 M Na_2_CO_3_	ZnO-Cu_2_O nanoparticles	CH4: 1080 μmol/g_cat_ h CO: 1.4 μmol/g_cat_ h	[70]
5 W UVC lamp	Aqueous suspension in 0.1 M Na_2_C_2_O_4_, 0.1 M KBrO_3_, 0.1 M NaOH or water	CuO	CH4: 1000 µmol/L-g CO: 6 µmol/L-g	[71]
300 W Xenon lamp	Aqueous suspension in 1M NaOH	Cu_2_O/CNPs	CH_3_OH: 236.43μmolg^−1^cat	[72]
300 W Xe arc lamp	Aqueous suspension in water	Ag-Cu_2_O/ZnO nanorods	CO: 9.94 μmol/g	[73]
1 kW high-pressure Hg (Xe) arc lamp	Aqueous suspension in water	Cu_2_O/TiO_2_	CO: 2.11 μmol g_cat_^−1^ h^−1^	[74]
100 W Xenon solar simulator with AM 1.5 filter	Powder photocatalyst with CO_2_	Cu_x_O-TiO_2_	CH4: 221.6 ppm/g-h	[75]
20 W white cold LED flood light	Aqueous suspension in DMF and water	rGO-CuO, rGO-Cu_2_O	rGO-CuO: CH_3_OH: 1228 μmol/g rGO-Cu_2_O: CH_3_OH: 862 μmol/g	[76]
200 W Xe lamp	Powder photocatalyst with CO_2_	TiO_2_-loaded Pt and Cu	Pt/TiO_2_: CH_4_: 11 µmol/g-h CO: 2.2 µmol/g-h Cu/TiO_2_: CH_4_: 8.7 µmol/g-h CO: 5.4 µmol/g-h Pt-Cu/TiO_2_: CH_4_: 9.8 µmol/g-h CO: 5.9 µmol/g-h	[77]
300 W Xe lamp	Aqueous suspension in water	Cu_2_O	CH_3_OH: 1.2 mol g^−1 ^h^−1^	[78]

**Table 3:** Summary of CO_2_ reduction using phthalocyanines.

Light Source	Reaction Medium and Electrolyte	Catalyst	Formate & Other Products	Ref.
300 UV-VIS UV-VIS lamp and 400 W Xe-Halogen lamp S: SANOLIUX HRC uv vis lamp 300 W H: RADIUM Xe-Halogen lamp 400 W	Aqueous suspension in water, pH adjusted by NaOH or H_3_PO_4_	Lipophilic phthalocyanines/TiO_2_ composites	Product yield in μmol/g cat TiO_2_ with pH 3 and a S/H light source: 131 HCOOH TiO_2_-H_2_Pc with pH 3 and a S/H light source: 75 HCOOH TiO_2_-CuPc with pH 3 and a S/H light source: 208.5 HCOOH TiO_2_-ZnPc with pH 3 and a S/H light source: 88.5 HCOOH TiO_2_-CuPc with pH 7 and a S/H light source: 63.4 HCOOH TiO_2_-CuPc with pH 13 and a S/H light source: 65.2 HCOOH TiO_2_-CuPc with pH 3 and a S light source: 32.6 HCOOH TiO_2_-CuPc with pH 3 and a H light source: 52.2 HCOOH	[79]
500 W tungsten-halogen lamp	Membrane dipped in water with 0.1 M TEA and 0.1 M HCIO_4_	MP-Nafion, MPC-Nafion	Nf/PP: 8 × 10^−5^ mol Nf/CoTPP: 2.9 × 10^−4^ mol Nf/FePC: 11 × 10^−5^ mol Nf/ZnPC: 2 × 10^−4^ mol	[80]
500 W tungsten–halogen lamp	Membrane dipped in 0.1 M triethanolamine and 0.1 M HClO_4_	MPC-Nafion	Nf-CoPc: 1.7 × 10^4^ mol Nf-ZnPc: 2.0 × 10^4^ mol	[81]
500 W tungsten–halogen lamp	Aqueous suspension in NaOH	CoPc-TiO_2_	With 0 [HCHO]/M and 0 [CH_3_OH]/M: HCOOH: 289.9 μmol/(g cat) With 0 [HCHO]/M and 0.5 [CH_3_OH]/M: HCOOH: 292.8 μmol/(g cat) With 0 [HCHO]/M and 5 [CH_3_OH]/M: HCOOH: 301.1 μmol/(g cat) With 0.1 [HCHO]/M and 0 [CH_3_OH]/M: HCOOH: 9731.3 μmol/(g cat) With 1 [HCHO]/M and 0 [CH_3_OH]/M: HCOOH: 82660.5 μmol/(g cat)	[82]
500 W tungsten-halogen lamp	Aqueous suspension in 0.1 N NaOH	CoPc-TiO_2_	1.0 wt% CoPc/TiO_2_: HCOOH: 450.6 μmol/g cat 0.7 wt% In-situ CoPc/TiO_2_: HCOOH: 1487.6 μmol/g cat	[83]
125 W high pressure mercury lamp	CO_2_ passed through catalyst-coasted reaction vessel	CuPc-TiO_2_	14% photoconversion	[84]
300 UV-VIS UV-VIS lamp and 400 W Xe-Halogen lamp	Aqueous suspension in water, pH adjusted by NaOH or H_3_PO_4_	CuPc-TiO_2_	HCOOH: 239.5 μmol/gcat	[85]

**Table 4:** Summary of CO_2_ reduction using metal-organic frameworks.

Light Source	Reaction Medium and Electrolyte	Catalyst	Formate & Other Products	Ref.
500 W Xe lamp	Aqueous suspension in 5:1 ratio of MeCN and TEOA	NH_2_-Uio-66(Zr)	NH_2_-Uio-66(Zr): HCOOH: 13.2 μmol in 10 h Mixed NH_2_-Uio-66(Zr): HCOOH:20.7 μmol in 10 h NH_2_-UiO-66(Zr): HCOOH: None Mixed NH_2_-UiO-66(Zr): HCOOH:7.28 μmol	[88]
300 W Xe lamp	Aqueous suspension in 5:1 ratio of MeCN and TEOA	MIL-101 (Fe), MIL-53 (Fe), MIL-88B (Fe),	NH_2_-MIL-101(Fe): HCOOH: 178 μmol MIL-101(Fe): HCOOH: 59.0 μmol NH_2_-MIL-53(Fe): HCOOH: 46.5 μmol MIL-53(FE): HCOOH: 29.7 μmol NH_2_-MIL-88(Fe): HCOOH: 30.0 μmol MIL-88(Fe): HCOOH: 9.0 μmol	[89]
500 W Xe lamp	Aqueous suspension in 5:1 ratio of MeCN and TEOA	NH_2_-MIL-125(Ti)	NH_2_-MIL-125(Ti): HCOOH: 8.14 μmol NH_2_-MIL-125(Ti) (λ > 450 nm): HCOOH: 3.83 μmol	[90]
470 nm LED	Aqueous suspension in 4:1 ratio of DMF/TEOA solvent mixture containing 0.2 M 1-benzyl-1,4-dihydronicotiamide (BNAH)	Mn(bpydc)(CO)_3_Br incorporated into Zr(IV)-based metal−organic framework	Turnover numbers for Products: UiO-67-Mn(bpy)(CO)_3_Br(b) for 4 h: HCOOH: 50 UiO-67-Mn(bpy)(CO)_3_Br(b) for 18 h: HCOOH: 110	[91]
300 W Xe arc lamp	Aqueous suspension in 4:1 mixed solution of acetonitrile (MeCN)-triethanolamine (TEOA), which contained 1-benzyl-1,4-dihydronicotiamide (0.1 M, BNAH)	Zr_4.3_Ti_1.7_O_4_(OH)_4_(C_8_H_7_O_4_N)_5.17_(C_8_H_8_O_4_N_2_)_0.83_	Zr_4.3_Ti_1.7_O_4_(OH)_4_(C_8_H_7_O_4_N)_5.17_(C_8_H_8_O_4_N_2_)_0.83_: HCOOH: 31.57 μmol UiO-66(Zr/Ti)-NH_2_: HCOOH: 4.66 µmol	[92]
